# Do savanna trees mast? Phenological dynamics of flowering and fruiting in savanna tree species

**DOI:** 10.1007/s00442-025-05706-3

**Published:** 2025-05-16

**Authors:** Corli Coetsee, Benjamin J. Wigley, Steven I. Higgins

**Affiliations:** 1 Savanna Node, Scientific Services, SANParks, Skukuza, 1350 South Africa; 2 School of Natural Resource Management, Nelson Mandela University, George Campus, George, 6530 South Africa; 3 Plant Ecology, University of Bayreuth, Universitaetsstrasse 30, 95447 Bayreuth, Germany

**Keywords:** African savanna, Evolutionary benefits, Phenology, Proximate causes, Reproductive events, Resource matching

## Abstract

A priori, it is not clear if masting should be expected in savannas and few studies have attempted to detect masting in savannas. We tracked the flower and fruiting phenology of 18 savanna woody species on a monthly basis in Kruger National Park for 8 years. We used multiple metrics to detect masting including phenological intensity and its CV, phenological volatility, synchrony and the proportion of failure years. Additionally, we used a process-based model of plant growth to test whether resource matching could explain the observed phenological behaviour. Overall, the measured masting metrics provided no unequivocal evidence for masting. For 4 of the 18 study species, the fruiting CV, synchrony and volatility were consistent with masting. The process-based model of plant growth could reproduce observed flowering and fruiting behaviour, suggesting that resource matching could explain the observed phenological behaviour of the species. We propose that future research should explore the possibility that masting may not be selected for in savannas due to the prevalence of generalist pollinators, dispersal agents and seed predators. Although masting does not appear to be a prevalent phenological strategy in savannas, we detected large between species variation in reproductive phenology, which is likely to have consequences for the trophic dynamics of the study system.

**Supplementary Information:**

The online version contains supplementary material available at 10.1007/s00442-025-05706-3.

## Introduction

Masting is when reproduction in iteroparous plants is characterised by occasional large synchronous reproductive events (Bogdziewicz et al. 2023; Qiu et al. 2023). The between-individual synchrony in reproductive effort characteristic of masting can be observed at large, sometimes continental-wide, scales (Ascoli et al. 2017). Masting is relatively common in ecosystems found at high or low latitudes such as tussock grasslands, temperate forests and boreal forests (Kelly and Sork 2002; Crone et al. 2009; Tanentzap et al. 2012; Pearse et al. 2016; Qiu et al. 2023). Masting is less reported in the tropics, but has been detected in the Dipterocarp forests of South East Asia (Visser et al. 2011; Schauber et al. 2002) and tropical far-northern Australia (Wright et al. 2022). Masting can increase the fitness of plants by increasing pollination success and/or allowing ecologically significant numbers of seeds to escape predation (Yamauchi 1996; Kelly and Sork 2002; Visser et al. 2011). While masting can enhance fitness in some species, it can also have profound trophic consequences for terrestrial ecosystems (Czeszczewik et al. 2020; Selonen et al. 2016; Clotfelter et al. 2007). Masting has also been reported to affect human health by stimulating respiratory allergies and promoting tick-borne diseases (Bregnard et al. 2021; Jones et al. 1998).

Masting may be observed for different reasons (Kelly and Sork 2002; Pearse et al. 2016). First, if masting provided fitness benefits, it should be selected for (an ultimate reason). Alternatively, it might be that masting is a simple mechanical consequence of variance in the resources and conditions that plants are exposed to (a proximate reason). Fitness benefits can be interpreted by invoking economy of scale (EOS) arguments (Pearse et al. 2016), where occasional large reproductive efforts yield, over the lifetime of a plant, more offspring than smaller, more regular efforts (Norton and Kelly 1988). The mechanism of EOS can involve predator saturation, dispersal efficiency, pollination efficiency, and environmental predictability. Seed predators can select for masting when larger seed crops are synchronised among individuals resulting in a lower proportion of seed predation (Janzen 1971; Silvertown 1980). During mast years, animal pollinators and dispersers are attracted by the large floral displays and fruit crops, leading to higher pollination and dispersal rates (dispersal efficiency hypothesis, Janzen 1970). However, if these animal pollination and dispersal agents are saturated by large floral and fruit crops, then reproduction rates may decrease, which would select against masting (Janzen 1971; Silvertown 1980; Herrera et al. 1998). Masting should be strongly selected for in wind-pollinated and self-incompatible species that can enhance pollination through synchronised above-average flowering effort (Nilsson and Wastljung 1987; Norton and Kelly 1988). Environmental prediction can create an EOS when, for example, species produce large seed crops immediately after stand replacing fires, allowing them to profit from high post-fire resource availability (e.g. high light, high nutrients, low competition) (He et al. 2016; Ascoli et al. 2020; Beck et al. 2024).

Proximate level hypotheses for masting have been comprehensively reviewed by Pearse et al. (2016). The pure proximate hypothesis is resource matching where a plant allocates a constant proportion of resources to reproduction, which means that in years with high resource availability reproduction is higher. A variation on this is resource switching. In resource switching, a plant switches allocation from growth to reproduction, and one of the ultimate selection mechanisms discussed above is needed to select for switching (Tanentzap and Coomes 2012; Moghaddam and Ende 2013; Miyazaki et al. 2002; Miyazaki 2013; Han and Kabeya 2017; Fernández-Martínez et al. 2019; Müller-Haubold et al. 2015). A further hypothesis is the research surplus hypothesis: resources surplus to growth, defence and maintenance needs are allocated to reproduction, with the environment allowing some years to generate higher surpluses than others.

There are very few reports of masting in savanna species. It is not clear whether this is due to a lack of multiple year phenological data (Wright et al. 2022) or because masting is rare in savannas. On one hand, it could be argued that selection for masting should exist in savannas. For example, predator saturation would enhance the fitness of savanna trees, as it is established that seed predation negatively impacts on the demography of savanna trees (Stevens 2021; Cilles et al. 2016). On the other hand, wind pollination is rare, and recruitment opportunities are relatively unpredictable, suggesting that the pollination and environmental predictability selective routes to masting are unlikely in savannas. Recent meta-analyses also suggest that masting is less prevalent in ecosystems, including savannas, where predator saturation is unlikely (Qiu et al. 2023; Zwolak et al. 2022). For example, Zwolak et al. (2022) in their meta-analysis of predator saturation and masting found that vertebrate seed predators were often not saturated, presumably because they have generalist diets and are relatively mobile, which means that their abundances are not drawn down in non-masting years. They also showed that masting is more likely to provide a net fitness benefit in species-poor plant communities because seed predators have more restricted opportunities to switch to other species during non-masting years. Qiu et al. (2023) suggest that mutualist dispersers (e.g. fleshy fruited species or nuts in cones) neutralise the benefits of masting for predator satiation and that trees with mutualist dispersers will avoid masting (i.e. have low volatility, low synchronicity). Taken together, the existing literature leaves us uncertain whether masting strategies are to be expected in savanna tree species.

In this study, we followed the phenological behaviour (leaf, flowering and fruiting) of 18 common savanna tree species over 8 years at three sites in a southern African savanna in the Kruger National Park (KNP). We use a range of descriptive statistics to evaluate whether the study species mast. The study system has a full set of biotic agents, including vertebrate dispersers, both vertebrate and invertebrate predators, which could allow both the predator saturation and the dispersal efficiency hypotheses to play out. We further used a resource matching model to test whether proximate factors can explain the observed reproductive phenology of the study species. The hypothesis is that if a resource matching model can predict the observed masting metrics, then a proximate explanation for any detected masting behaviour would exist and selection (i.e. ultimate causes) for masting need not be invoked.

## Materials and methods

### Study site

The Kruger National Park covers nearly 20 000 km^2^ (22° 20ʹ to 25° 30’ S, 31° 10’ to 32° 00’ E) in South African ‘lowveld’ savannas (elevation 260–839 m). Phenology was monitored at three sites in relative close proximity (one less than 1 km and the other around 10 km away) to the main administrative centre, Skukuza (see Table 1 for locations). Data from the Skukuza weather station indicate a long-term rainfall average of 550 mm (Venter et al. 2003). Rainfall is seasonal and variable, falling mainly during the austral summer between October and April (Venter et al. 2003). All sites were situated on granite which is characterised by oxisols (Fey 2010); however, finer-scale complexity exists, for instance nutrient-rich patches (duplex soils) can be found on the bottomlands and on sodic midslopes (Venter et al. 2003). Vegetation is classified as Granite Lowveld which is mostly open woodland on the uplands dominated by *Combretum* spp. and dense thickets to open woodlands on the bottomlands and sodic sites, dominated by *Acacia nigrescens*, *Dichrostachys cinerea* and *Grewia bicolor* (Mucina and Rutherford 2006). At seeplines, where uplands meet bottomlands, a dense fringe of *Terminalia sericea* is often found.

**Table 1:** Species monitored at each of the three study sites

Species	Code	Site(s)	Fruit type	Pollinator	Dispersal agent
*Acacia gerrardii*	acager	Su	Dry pod, dehiscent	Insect, wind	Zoochory/barochory
*Acacia grandicornuta*	acagra	Sk	Dry pod, dehiscent	Insect	Zoochory/barochory
*Acacia exuvialis*	acaexu	Nk, Sk	Dry pod, dehiscent	Insect	Barochory
*Acacia nigrescens*	acanig	Nk, Sk, Su	Dry pod, dehiscent	Insect	Zoochory/barochory
*Acacia nilotica*	acanil	Su	Dry pod, indehiscent	Insect	Zoochory/barochory
*Acacia tortilis*	acator	Sk	Dry pod, indehiscent	Insect	Zoochory/barochory
*Balanites maughamii*	balmau	Sk	Fleshy	Insect	Zoochory
*Combretum apiculatum*	comapi	Nk, Sk	Dry samara, indehiscent	Insect	Anemochory
*Combretum hereroense*	comher	Nk, Su	Dry samara, indehiscent	Insect	Anemochory
*Combretum zeyheri*	comzey	Su	Dry samara, indehiscent	Insect	Anemochory
*Grewia bicolor*	grebic	Sk	Fleshy	Insect	Zoochory
*Grewia flavescens*	grefla	Nk, Sk	Fleshy	Insect	Zoochory
*Kigelia africana*	kigafr	Sk	Fleshy	Bird/bat	Zoochory
*Lannea schweinfurthii*	lansch	Su	Fleshy	Insect	Zoochory
*Pappea capensis*	papcap	Nk, Sk	Fleshy	Insect	Zoochory
*Sclerocarya birrea*	sclbir	Nk, Sk	Fleshy	Insect	Zoochory
*Terminalia sericea*	terser	Nk, Su	Dry samara, indehiscent	Insect	Anemochory
*Ziziphus mucronata*	zizmuc	Nk, Su	Fleshy	Insect	Zoochory

The site names are abbreviated as Nk (Nkuhlu), location − 24.9808, 31.78026; Sk (Skukuza), location − 24.9851, 31.585; and Su (Supersite, Smit et al. 2013), location − 24.9938, 31.5927. Code names used in all figures and information on fruit type, pollinator and dispersal agents are provided (Miller 1996; Olofsson and Strengbom 2000; Coe and Coe 1987; Midgley et al. 2015; Helm et al. 2011; Favaretto et al. 2024; Namah et al. 2019; Veereshkumar et al. 2021). Apart from *Pappea capensis*, all species are deciduous

### Data collection

In August 2015, we tagged 10 individuals each of 18 common species distributed across three sites (Table 1). Not all species were present at all sites; if a species was present at a site 10 individuals of this species were monitored (a total of 290 individuals). Species nomenclature is based on Palgrave (2015). Very little is known about the pollination of these species; many of the study species appear to be pollinated by generalist insect pollinators, including bees and scarab beetles (Mawdsley and Sithole 2010). It has been suggested that giraffe pollinates *A. nigrescens* and that this is somehow associated with the dry season flowering of *A. nigrescens* (Du Toit 1990), although this interpretation has been challenged (Fleming et al. 2006). *Kigelia africana* is pollinated by birds (Newman et al. 2021; Namah et al. 2019) and bats (Namah et al. 2019).

In the figures, we refer to the 18 species by the first three letters of the genus and species names (see Table 1). Tagged trees died occasionally; if a tree was killed during a year cycle (August to July), no further measurements were taken. Lost trees were replaced every August and the new tree was given a new identifier.

All sampled individuals were in the upper quantile of plant sizes for that species, allowing us to assume that size effects on variance in phenological behaviour did not bias our data or analyses. The tagged trees were inspected monthly by two observers and amount of leaf cover, flower cover and fruit cover were assigned to one of five ordinal categories. The categories are based on the Fournier intensity percentage method (Fournier 1974). The ordinal categories used were 0: absence of the phenophase; 1: 1–25%; 2: 26–50%; 3: 51–75%; and 4: 76–100% of the magnitude of the phenophase present in relation to the maximum volume of the canopy). Although there has been some turnover of observers over the project lifetime, all were trained by the lead author. The same two observers have been conducting the surveys since mid-2019.

### Descriptive statistics

Studies of masting often use the coefficient of variation (CV) of interannual seed production to quantify the degree of masting—the standard deviation of annual seed crop divided by the mean of the annual seed crop (Kelly and Sork 2002; Wright et al. 2005; Kelly 1994; Herrera et al. 1998). The CV has the advantage of being a dimensionless number, which allows for a comparison among species with significantly different mean and standard deviation values. High CVs (> 1) of yearly seed crop sizes over time are typically used to diagnose masting (Kelly 1994). However, although masting would lead to high CVs of seed production, it cannot unequivocally diagnose masting. This is because it does not quantify differences in the temporal dependency of seed production typical of masting syndromes. Moreover, high CVs can arise from species that fail to reproduce in some years, but never produce sufficiently large seed crops to induce EOS benefits typical of masting syndromes (Crone et al. 2011; Bogdziewicz et al. 2023). To address the limitations of CV as a measure of masting, we additionally calculated the following masting metrics.

1. **Phenological intensity** is simply the mean abundance (represented by ordinal class intensity) of leaves, flowers or seeds produced in a phenological year.
2. **Phenological volatility** is a metric that increases with variation in phenological intensity and with lags in phenological activity. Volatility better accommodates zeros and lags in the data than CV does. Volatility is calculated using the R function mastif::mastSpectralDensity (Clark et al. 2019). Since volatility is highly sensitive to the units being used to measure phenological intensity, the values are not necessarily comparable between studies.
3. **Synchrony** is a metric that describes the synchrony of phenological intensity metrics between individuals, it was calculated as the mean Pearson’s cross-correlation with all other trees of the same species in the study. In previous work, synchrony metrics > 0.2 has been used to diagnose masting (Bogdziewicz et al. 2019).
4. **Proportion failure years** is the proportion of years in which the phenological intensity is zero. The failure rate is a potential indicator of masting strategies, although species with high observed failure rates might simply be low fecundity species.

We additionally calculated the day of year (DOY) in which phenological activity was observed. This is calculated as the mean and standard deviation of DOY when the observed intensity is greater than the mean observation intensity (calculated using circular statistics). Principal components analysis (PCA) was used to explore the variation in the masting metrics (i.e. CV, failures, intensity, synchrony and volatility) among the 18 study species. The PCA was performed using the mean values of the masting metrics for each species using the R function stats::prcomp (R Core Team 2023).

### Resource matching model

We used a process based model of plant growth to test whether resource matching can explain the observed phenological behaviour. To calibrate the model we used a Bayesian state space approach. See Auger-Méthé et al. (2021) for an introduction to state space modelling in an ecological context. The process model underlying the state space analysis is described in Higgins et al. (2025) and in supplementary materials S1 and S2. Briefly, the process model considers how biomass and substrate pools accumulate over time as influenced by assimilation, growth and allocation processes. These process rates are forced by climate reanalysis data (ERA5-Land data, European Centre for Medium-Range Weather Forecasts Reanalysis v. 5; hereafter, ERA5, see S2.1 for more detail) (Hersbach et al. 2020; Muñoz-Sabater et al. 2021) and therefore articulates a purely resource based hypothesis of phenological behaviour. Conceptually, the analysis takes the form:

1 $$ {\mathbf{X}}_{t} = \Delta ({\mathbf{X}}_{{t - {1}}} ,\theta ) + \in $$

2 $$ \mu_{j,t} = \delta_{j} - {\mathbf{X}}_{t} \beta $$

3 $$ Q_{t} = {\text{invlogit}}(\mu_{t} ) $$

4 $$ P_{1,t} = Q_{1,t} $$

5 $$ P_{j,t} = |Q_{1,t} - Q_{j - 1,t}| \; {\text{and}}\; j = {2},..(J - {1}) $$

6 $$ P_{j,t}= {1} - Q_{j - 1,t} $$

7 $$ {\mathbf{y}}_{i,t}\sim {\text{CAT}}(P_{i,1:J,t}). $$

Here, **X**_*t*_ is a matrix containing the growth model’s state at time *t* and *ϵ* is the process error.

∆(**X**_*t−*1_*, θ*) represents the state space model as influenced by the *θ* parameters described in equations S15 to S18. The priors on the *θ* parameters are give vague normal priors. The process errors represented by *ϵ* are assumed to be normal random variables centred on zero. Each state variable *MS, MR, CS, CR, NS, NR* (see equations S1-S14) has an associated process error term. The priors for the variances of the process error terms are assumed to follow the gamma distribution.

For this analysis **X** considers only one predictor variable for each phenological observation type. Specifically carbon assimilation *U*_*C*_ (equation S7) is used to predict the leaf phenology and the substrates, calculated as

$${\frac{1}{f_C} \frac {CS+CR}{MS+MR} }+ {\frac{1}{f_N} \frac{NS+NR}{MS+MR}}$$

are used to predict the flowering and fruiting phenology. The substrate influence on flowering and fruiting phenology act with a temporal lag (*λ*) and the temporal lag differs for flowering and fruiting phenology. The priors of the lag parameters are uniform between 0 and 52 weeks.

The carbon assimilation and lagged substrate variables are then used as predictor variables in an ordinal logistic regression model. Here, *µ* is the linear predictor in the logistic regression and *δ* is a vector of intercepts, describing the *J* ordinal classes (*j* = 1*, …,* (*J* − 1)). In this study, there are five classes. We assume that *δ* have vague normal priors. Similarly, *β* is a vector of coefficients describing the effect of each of the *K* state variables in **X** on the linear predictor *µ*. Vague normal priors are used for *β*. The notation CAT represents the probability density function of the categorical distribution. *P* describes a vector of length *J* describing the probabilities of being in each of the *J* possible phenological classes where Σ*P* = 1.

The vector **y** contains the phenological observations made for tree *i* at each point in time *t*. The state space model is run on a weekly time step, whereas we only evaluate the model at time points when observations were made. Linear interpolation is used to estimate the state space variables at the observation time points. To simplify the notation, this is not explicitly represented in Eqs. 1–7.

We estimate this model for each species separately. That is, we assume that each species has one set of *δ*, *β*, *λ* and *θ* parameters that can explain its leaf, flower and fruiting phenology across all sites. This ensures that all phenological observations influence the estimation of the growth model’s *θ* parameters; that is, the model attempts to identify a single vector of *θ* parameters that produce a set of hidden states (**X**) that can predict all three classes of phenological observations at all sites. Supplementary section S3 provides a list of the posterior estimates of the parameters, which further clarifies which parameters are estimated.

The state space model is formulated using the R-package LaplacesDemon (Statisticat-LLC 2020). The model also requires the parameterisation of the initial plant state, which is also given a vague uniform prior. The effects of initial conditions are, however, unimportant for the interpretation of this analysis. This is because we run the model from the year 2000 and the observation data begin in the year 2015, by which time any effects of initial conditions are no longer detectable. We used the DRAM MCMC algorithm as implemented in LaplacesDemon (Statisticat-LLC 2020) and its default control parameters, to estimate the posterior distributions of the model parameters. We calculated the effective sample size (ESS) for each model parameter as implemented in LaplacesDemon (Statisticat-LLC 2020). ESS estimates how the sample size is reduced by correlation in the Markov chain and is a widely used convergence diagnostic (Roy 2020). We ran chains until all parameters had an ESS > 50. To assess the model’s ability, we calculated the prediction accuracy. A correct prediction is when the predicted class exactly matches the observed class within the ordinal scale of the data (we have five ordinal classes). We further fitted the model using the R function DEoptim::DEoptim (Mullen et al. 2011) to verify that the MCMC algorithm had not missed important regions of the parameter space (results not shown). DEoptim implements the differential evolution algorithm (Storn and Price 1997), which is a robust genetic algorithm that is known to perform stably on high dimensional and multi-modal problems (Ardia et al. 2011).

## Results

**Fig. 1:**
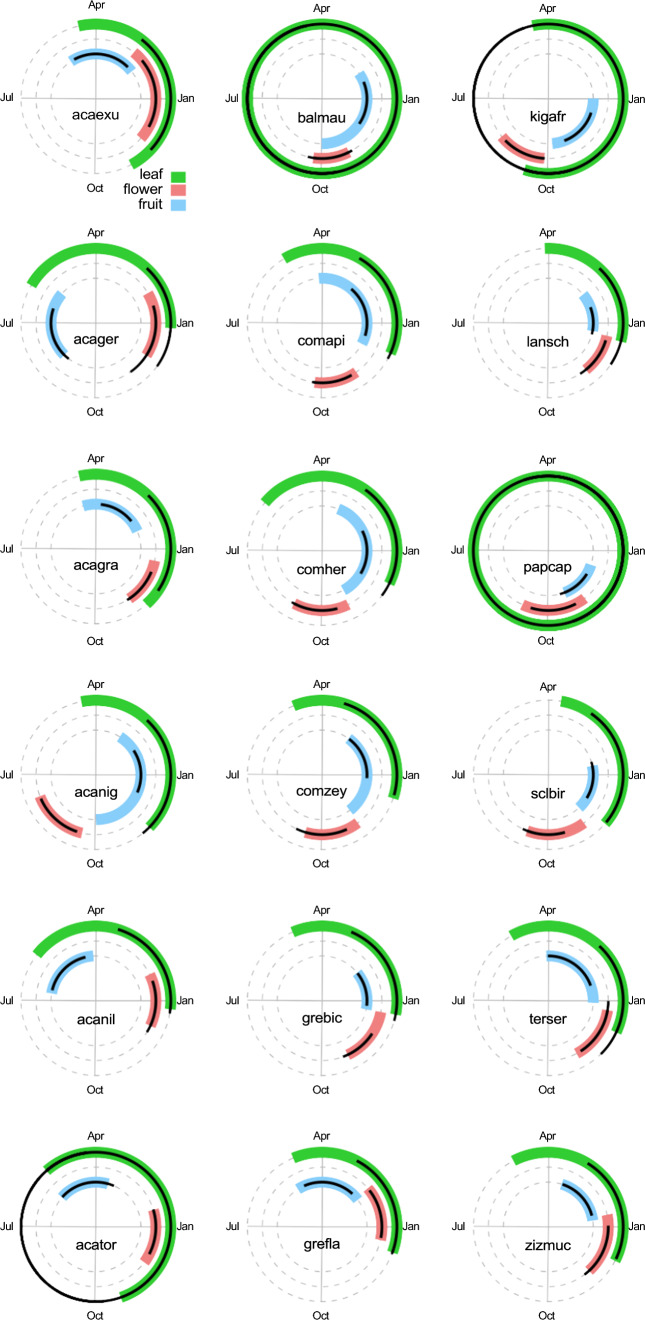
Timing of phenological events for 18 savanna tree species. The phenological event timing is estimated as 1.5 standard deviations spanning the mean day of year of observations of the phenological event (i.e. representing the most intense period of the phenological activity). The black lines are the model predictions

For the majority of the studied species, flowering was centred at the beginning of the wet growing season (Fig. 1). The rains at our study site typically start towards the end of October (DOY 298, 25 October) (Wigley et al. 2024). A few species, such as *Acacia nigrescens* and *Kigelia Africana,* flower before the start of the rains, and some later, around the summer solstice, but a fair number appear to time their most intense flowering to coincide with the onset of the rainy season (e.g. *Balanites maughamii* DOY 284, *Combretum apiculatum* DOY 289, *Combretum hereroense* DOY 274, *Combretum zeyheri* DOY 285, *Pappea capensis* DOY 281, and *Sclerocarya birrea* DOY 283). The culmination of fruiting is more variable. Although the core summer months are used by many species (e.g. *Balanites maughamii, Lannea schweinfurthii, Combretum hereroense, Acacia nigrescens, Combretum zeyheri, Sclerocarya birrea, Grewia bicolor*), the peak fruiting of some species are centred at the end of the summer (e.g. *Acacia grandicornuta, Acacia tortilis, Ziziphus mucronata*). In a few species (e.g. *Acacia gerrardii, Acacia nilotica*) fruiting is centred into the dry winter period. It should be noted that the coloured lines on Fig. 1 do not indicate the absolute start and end of leaf, flower and fruit displays, but indicate 1.5 standard deviations of the estimated mean day of year of the leaf, flower and fruit displays.

**Fig. 2:**
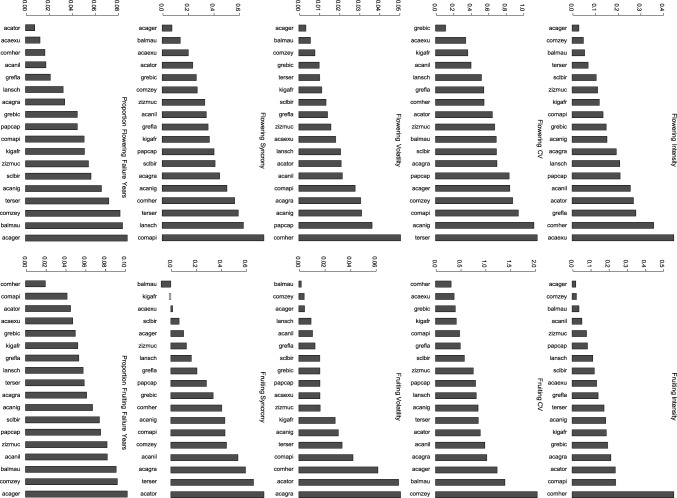
Flowering and fruiting phenology metrics of 18 savanna tree species, including intensity, coefficient of variation (CV), volatility, synchrony and failure years

The mean flowering intensity over the study period varied between 0.03 for *Acacia gerrardii* and 0.36 for *Combretum hereroense* (Fig. 2). The mean fruiting intensity varied between 0.02 for *Acacia gerrardii* to 0.56 for *Combretum hereroense*. *Combretum hereroense* had a conspicuously higher fruiting intensity than the other species in our study. The CV of flowering intensity was for most species below 1, with only *Acacia nigrescens* and *Terminalia sericea* marginally exceeding 1. The fruiting CVs were higher than flowering CVs, and here the four species with CVs above 1 included *Acacia gerrardii, Acacia grandicornuta, Balanites maughamii* and *Combretum zeyheri*. Flowering volatility was evenly distributed over the study species (ranging from 0.003 for *Acacia gerrardii* to 0.05 for *Combretum zeyheri*), whereas there were low (below 0.20), medium (0.20–0.40) and high (> 0.04) clusters in fruiting volatility, with *Acacia grandicornuta, Acacia tortilis* and *Combretum hereroense* being the most volatile species. Both flowering and fruiting synchronies were evenly distributed over the study species. All but two of our species had flowering synchrony of above 0.20 (with 8 species above 0.4) and 11 species had fruiting synchrony of above 0.20 (and 8 above 0.40). The flowering and fruiting failure rates were also evenly distributed among species with failure rates covering the range of 0.01 to 0.09. *Acacia gerrardii, Balanites maughamii* and *Combretum zeyheri* had the highest flowering and fruiting failure values (0.08–0.09 and 0.09–0.10, respectively).

**Fig. 3:**
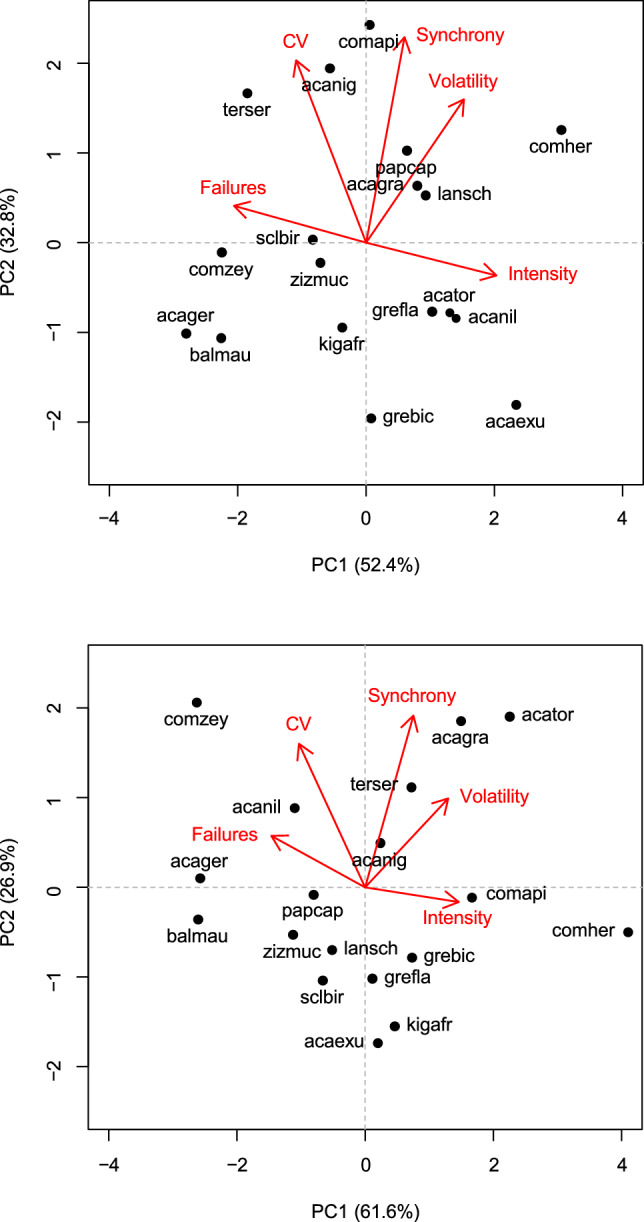
Principal component analysis of flowering and fruiting phenology metrics of 18 savanna tree species

We used the metrics described in the previous paragraph to conduct a principal components analysis. The first principal component axis explained 52% of the variance in the flowering data and separated species with high levels of failures and low flowering intensities from those with low failure rates and high flowering intensities. For example, *Combretum hereroense* had a low failure rate and high intensities of flowering (Fig. 3). The second principal component axis explained an additional 33% of the variance and was associated with species of high CV, synchrony and volatility. *Combretum apiculatum, Acacia nigrescens* and *Terminalia sericea* were species with high flowering CV, synchrony and volatility.

The fruiting data PCA yielded a qualitatively similar biplot to the flowering PCA (Fig. 3). The first principal component explained 62% of the variance in the fruiting data, while the second principal component axis explained an additional 27%. *Acacia grandicornuta, Acacia tortilis, Combretum zeyheri* and *Terminalia sericea* were species with high fruiting CV, synchrony and volatility.

The ability of the calibrated resource-based model to reproduce the observed patterns in the timing of leaf, fruit and flower display is shown by the black lines in Fig. 1. The prediction accuracy was modest for leaf observations (across species range = 0.22–0.55; mean = 0.34), but good for flower (range = 0.72–0.97; mean = 0.87) and fruit observations (range = 0.58–0.98; mean = 0.87). For flowering and fruiting, the model predicted the timing well in the sense that there was always an overlap between the observed and modelled timing of the events.

**Fig. 4:**
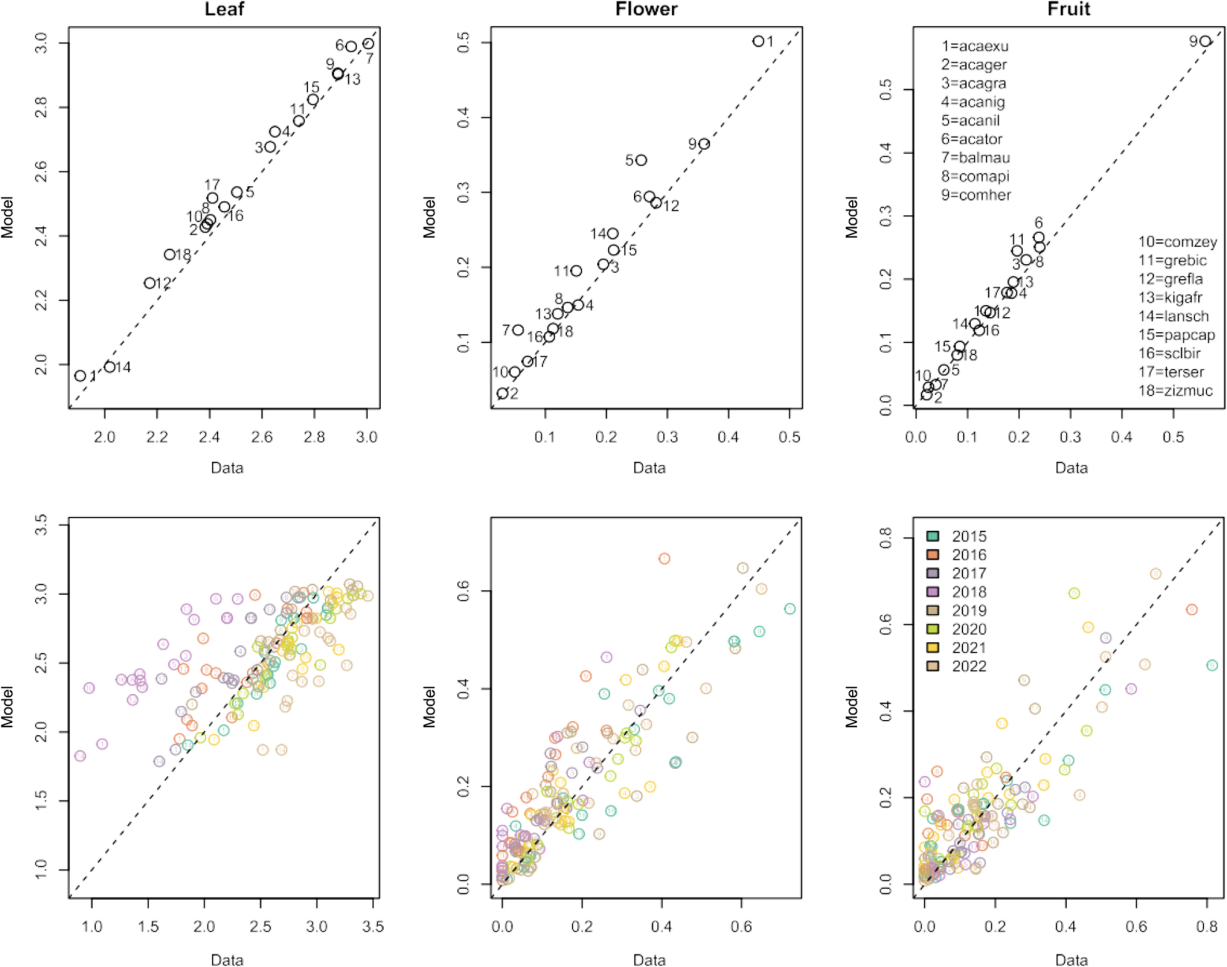
Modelled and observed leaf, flower and fruit phenology metrics of 18 savanna tree species. Each plotted point represents the mean intensity of leaf, fruit and flower phenology observed and predicted for a species. In the upper panels these are the means over all years, and in the lower panels the mean for each year

The ability of the model to reproduce the observed leaf, flower and fruit phenology data is summarised in Fig. 4. This illustrates that the model was able to reproduce the mean intensity of the observed intensities of leaf, flower and fruit displays of the 18 species without significant bias (upper panels). When we examine the displays predicted by the models in the different phenological years (2015–2022), it is clear that the between-year variation in leaf displays is not reproduced well (with most of the 2018 points above the 1:1 line). This is primarily due to year 2018 being rather poorly captured for all species. The ability of the model to predict the flower and fruit displays in the different years was, in contrast, better and relatively unbiased: the variation around the 1:1 line (Fig. 4) appeared unstructured (i.e. the residuals appear balanced).

## Discussion

Although masting has been reported from many different ecosystems, little work has considered this phenomenon in tropical savannas (Mduma et al. 2007; Wright et al. 2022). This study sought to detect masting using observations of both flowering and fruiting behaviour over 8 years for 18 species in a semi-arid savanna. To detect masting, we used a combination of metrics that have been used in previous studies (Bogdziewicz et al. 2023). Species with high CVs (CV > 1) of flowering and fruiting intensity included *Acacia nigrescens* and *Terminalia sericea* for flowering, and *Acacia gerrardii, Acacia grandicornuta, Balanites maughamii, Combretum zeyheri* for fruiting. The most volatile species included *Combretum hereroense* for flowering, and *Acacia grandicornuta* and *Acacia tortilis* for fruiting. The most synchronous species included *Combretum apiculatum* and *Lannea schweinfurthii* for flowering and *Acacia tortilis* and *Terminalia sericea* for fruiting.

*Combretum apiculatum, Acacia nigrescens* and *Terminalia sericea* were species with a combination of high flowering CV, synchrony and volatility. *Acacia grandicornuta, Acacia tortilis, Combretum zeyheri* and *Terminalia sericea* were species with high fruiting CV, synchrony and volatility. The discrepancies between flowering and fruiting are not surprising as species with very predictable year to year flowering can still have highly variable fruiting (Pearse et al. 2016; Montesinos et al. 2012; Mduma et al. 2007) created through mechanisms including delayed fertilisation (Satake and Kelly 2021), pollination limitation (Pearse et al. 2016) or flower abortion (Pearse et al. 2015). Our flowering and fruiting metric analyses therefore suggest that only 4 of the 18 study species (*Acacia grandicornuta, Acacia tortilis, Combretum zeyheri, Terminalia sericea*) were potentially masting species.

Although the masting metrics did not provide unequivocal evidence for masting, a PCA of the masting metrics revealed that species were overdispersed in the PCA biplots. This overdispersion finding agrees with Herrera et al. (1998) who suggested that masting expression falls on a continuum. A potential exception to this was observed in fruiting volatility where there was some evidence that species formed low, mid and high volatility groups (Fig. 2). Further evidence against masting in our study species is suggested by the negative relationship between failure and intensity for both flowering and fruiting (Fig. 3). In masting species, we would expect the intensity to remain constant or even increase despite high failure rates, as we would expect masting species to fail to reproduce in most years accompanied by high intensity fruiting and flowering events in other years.

We further found that a resource based model of plant growth, forced by reanalysis of climate system data, could reproduce the flowering and fruiting patterns of our study species (Figs. 1 and 4). Irrespective of whether the observed phenology metrics are sufficient to diagnose masting, the model’s ability to reproduce the phenological patterns suggests proximate rather than ultimate (evolutionary) reasons for the variance in the observed phenological behaviour may exist (Isagi et al. 1997). There are, however, two caveats that should be taken into account before jumping to this conclusion. First, we link the mechanistic growth model in a purely statistical way to the observed ordinal scale data. That is, there is no conservation of matter considered in the modelling of the phenological behaviour. Second, substrates are linked to the phenological behaviour with lags (the *λ* parameters referred to in the section describing the resource matching model). In our study, these lags have no explicit biophysical interpretation. While it is possible that future studies might propose a biophysical explanation, it might be that explaining the lags would require invoking ultimate explanations. Despite these caveats, the model may provide a useful foundation for future work, since it includes features previous authors have called for, such as the consideration of both carbon and nitrogen substrates and how they are allocated between substrate sources and sinks (Han and Kabeya 2017; Fernández-Martínez et al. 2019; Miyazaki 2013). Moreover, previous studies have modelled flowering and fruiting behaviour on an annual time step (e.g.Isagi et al. 1997; Monks et al. 2016), which potentially over-simplifies the relationships between environmental forcing, growth and substrate accumulation; the model used here, by modelling on a weekly time step, provides a convenient framework for considering intra-annual dynamics.

The limited evidence for masting found in this study raises the question of whether masting is expected in savanna ecosystems. Although masting is widespread at the global level, it tends to be more frequently associated with particular ecosystems and environments. Much of the previous work on masting in savannas has focussed on temperate savannas, often species-poor and mostly wind pollinated (Demeny et al. 2024; Zlotin and Parmenter 2008; Koenig and Knops 2005; Koenig et al. 2015; Parmenter et al. 2018), which reflects a global pattern of masting being more prevalent in clades inhabiting temperate latitudes where wind-pollination is common (Pearse et al. 2020). One of the few studies investigating masting in African savannas (Mduma et al. 2007) found that only 2 of 15 study species exhibited behaviour consistent with masting. Tropical environments are often characterised by high plant productivity, low year-to-year climatic variability, and animal pollination and dispersal. All of these factors would according to Kelly and Sork (2002) select against masting as the fitness benefits of higher seed production would be lower than the costs. For example, Herrera et al. (1998) and Qiu et al. (2023) proposed that fleshy fruited species that have their seeds dispersed by mutualistic animal dispersers should be masting avoiders, as there is no need to satiate predators through large synchronised fruiting events.

In African savannas, animal dispersal of fleshy fruits or nutritious pods may decrease seed predation. For example, endozoochory by large vertebrate herbivores decreases infestation by bruchid beetles, since seeds are often dispersed before infestation by bruchids can occur and because bruchids already in the seedpods are killed as they move through the digestive tract (Or and Ward 2003). Selection for such’biocontrol by endozoochorous dispersers’ will depend on the quantitative details. So while Mduma et al. (2007) suggested that *Acacia tortilis* synchronises the ripening and dropping of its seed pods to attract endozoochorous dispersers, the levels at which predators versus mutualistic dispersers become saturated are critical. The fitness benefits would be maximised when the seed predators are saturated, but the dispersal agents are not. That is, this strategy seems to require an intermediate level of masting expression.

In conclusion after 8 years of monitoring 290 individual trees spread across 18 species and 27 840 observations, we found limited evidence for masting in savanna tree species. Nonetheless, by using a range of metrics to describe fruiting and flowering behaviour, we show considerable variation in reproductive behaviour among species. This variation suggests potentially important niche differences, where we define the niche (following Chase and Leibold 2003) to include both the requirements of species (e.g. for pollinators or seed predators) and the impacts of species on their environment (e.g. on pollinators or seed predators). As pollinators, seed dispersers and seed predators are often large herbivores in tropical savannas, new axes of variation should be considered when studying trophic dynamics. Future work should consider whether complex indirect interactions between plants and animals, which at the same time drive selection against masting, may drive ecosystem resilience in tropical savannas (Mittelman et al. 2022).

## Supplementary Information

Below is the link to the electronic supplementary material.

Supplementary file1 (DOCX 33 KB)

## Data Availability

The datasets used and/or analysed during the current study are available from the corresponding author on reasonable request.

## Code availability

The code used during the current study is available from the corresponding author on reasonable request.
