# Correction: Do savanna trees mast? Phenological dynamics of flowering and fruiting in savanna tree species

**DOI:** 10.1007/s00442-025-05741-0

**Published:** 2025-06-06

**Authors:** Corli Coetsee, Benjamin J. Wigley, Steven I. Higgins

**Affiliations:** 1 Savanna Node, Scientific Services, SANParks, Skukuza, 1350 South Africa; 2 School of Natural Resource Management, Nelson Mandela University, George Campus, George, 6530 South Africa; 3 Plant Ecology, University of Bayreuth, Universitaetsstrasse 30, 95447 Bayreuth, Germany

**Correction: Oecologia (2025) 207:85** 10.1007/s00442-025-05706-3

In the original version of this article, the equations from 4–7 were incorrectly published. The old incorrect and the corrected version of the equations are given below.

Incorrect version:

4 $$ P1,t = Q1,t $$

5 $$ Pj,t = |Q1,t - Qj - 1,t|{\text{and}}j = 2,..(J - 1) $$

6 $$ Pj,t = 1 - Qj - 1,t $$

7 $$ {\mathbf{y}}i,t\sim {\text{CAT}}(Pi,1:J,t). $$

Corrected version:

4 $$ P_{1,t} = Q_{1,t} $$

5 $$ P_{j,t} = |Q_{1,t} - Q_{j - 1,t} |\;{\text{and}}\;j = 2,..(J - 1) $$

6 $$ P_{j,t} = 1 - Q_{j - 1} ,t $$

7 $$ {\mathbf{y}}_{i,t} \sim {\text{CAT}}(P_{i,1:J,t} ). $$

In the sentence beginning ‘For this analysis **X** considers only…’ in this article, the formula is displayed incorrectly as

^1CS+CR^

$$ {\text{C 1 NS}} + {\text{NR fN MS}} + {\text{MR}}, $$

when it should have been

$$ \frac{1}{{f_{C} }}\frac{CS + CR}{{MS + MR}} + \frac{1}{{f_{N} }}\frac{NS + NR}{{MS + MR}} $$

The original article has been corrected.

