# Supplementary material for: Do savanna trees mast? Phenological dynamics of flowering and fruiting in savanna tree species
Source: Oecologia. 2025 May 16;207(6):85. doi: 10.1007/s00442-025-05706-3 (PMC12084283; doi:10.1007/s00442-025-05706-3)
Supplement: Supplementary file 1 — Supplementary file1 (DOCX 33 KB) [file 442_2025_5706_MOESM1_ESM.docx]

# 684 Supporting Information

685 **S1 Plant growth model without environmental forcing**

686 The model described below is implemented in the R package TTR.PGM ([Higgins et al.](#_bookmark3),

687 [2025](#_bookmark3)). In the sections below we provide a summary, making it explicit which model variant

688 we used in this study. The model without environmental forcing closely follows the original

689 description of the Thornley Transport Resistance (TTR) model ([Thornley](#_bookmark2), [1998](#_bookmark2)). The shoot

690 and root mass pools (*MS* and *MR*; kg of structural dry matter) change as a function of

691 growth (*G_S_*, *G_R_*) and loss (*L_S_*, *L_R_*) processes,

*MS*[*t* + 1] = *MS*[*t*] + *G_S_*[*t*] *− L_S_*[*t*]*,* (S1)

*MR*[*t* + 1] = *MR*[*t*] + *G_R_*[*t*] *− L_R_*[*t*]*.* (S2)

692 Following [Thornley](#_bookmark2) ([1998](#_bookmark2)), *G_S_*, *G_R_*, *L_S_* and *L_R_* are defined as

*G_S_* = *g*

*CS NS MS*

*,* (S3)

*G_R_* = *g*

*CR NR*

*MR MS*

*,* (S4)

*L_S_* = *d*

1 +

*,* (S5)

*M*

*K*

*MS*

*L_R_* = *d*

*K .* (S6)

*M*

*MR*

1 +

*MR*

693 These equations specify that growth (*G_s_* and *G_r_*, units kg.day*−*1) varies as a function of the

694 carbon and nitrogen concentrations. *CS, CR, NS* and *NR* are the amounts (units kg) of

695 carbon and nitrogen in the roots and shoots, and *g* is the growth coefficient (units [kg C . kg

696 N . kg M*−*2]*^−^*^1^ . day*−*1), *d* is a biomass loss rate (units kg.kg*−*1.day*−*1). The parameter *K_M_*

697 (units kg) describes how loss varies with mass (*MS* or *MR*).

698 Carbon uptake *U_C_* is determined by the net photosynthetic rate (*a*, units kg.kg*−*1.day*−*1)

699 and the shoot mass (equation [S7](#_bookmark10)). Similarly nitrogen uptake (*U_N_* ) is determined by the ni-

700 trogen uptake rate (*b*, units kg.kg*−*1.day*−*1) and the root mass (equation [S8](#_bookmark11)). The parameter

701 *K_A_* (units kg) forces both photosynthesis and nitrogen uptake to be asymptotic with mass.

702 The second terms in the denominators of equations [S7](#_bookmark10) and [S8](#_bookmark11) model product inhibitions

703 of carbon and nitrogen uptake respectively, i.e. the parameters *J_C_* and *J_N_* (units kg.kg*−*1)

704 mimic the inhibition of source activity when substrate concentrations are high,

*UC* = (1 + *^MS^* )(1 + *^CS^* ) *,* (S7)

*aMS*

*K_A_ MS J_C_*

*bMR*

*UN* = (1 + *^MR^* )(1 + *^NR^* ) *.* (S8)

*K_A_ MR J_N_*

705 The substrate transport fluxes of C and N (*τ_C_* and *τ_N_* , units kg.day*−*1) between roots and

706 shoots are determined by the concentration gradients between root and shoot and by the

707 resistances. In the original model description ([Thornley](#_bookmark2), [1998](#_bookmark2)) these resistances are defined

708 flexibly, but we simplify and assume that they scale linearly with plant mass,

*τ* =  *MS MR*  *CS*

*C*

*MS* + *MR*

*MS*

*MR*

*− CR* *,* (S9)

*τ* =  *MS MR*  *NR − NS* *.* (S10)

*N*

*MS* + *MR*

*MR*

*MS*

709 The changes in mass of carbon and nitrogen in the roots and shoots are then,

*CS*[*t* + 1] = *CS*[*t*] + *U_C_*[*t*] *− f_C_G_s_*[*t*] *− τ_C_*[*t*]*,* (S11)

*CR*[*t* + 1] = *CR*[*t*] + *τ_C_*[*t*] *− f_C_G_r_*[*t*]*,* (S12)

*NS*[*t* + 1] = *NS*[*t*] + *τ_N_* [*t*] *− f_N_ G_s_*[*t*]*,* (S13)

*NR*[*t* + 1] = *NR*[*t*] + *U_N_* [*t*] *− f_N_ G_r_*[*t*] *− τ_N_* [*t*]*,* (S14)

710 where *f_C_* and *f_N_* (units kg.kg*−*1) are the fractions of structural carbon and nitrogen in dry

711 matter.

# 712 S2 Adding environmental forcing to the plant growth model

713 In this section we describe how the net photosynthetic rate (*a*), the nitrogen uptake rate (*b*),

714 the growth rate (*g*) and the loss rate (*d*) are influenced by environmental forcing factors. All

715 other model parameters are treated as constants. The parameters *a, b* and *g* are co-limited

716 by environmental factors in a manner analogous to Liebig’s law of the minimum, which is

717 a crude but pragmatic abstraction.

718

719

720

721

722

723

724

725

726

727

We use the Farquhar model of photosynthesis ([Farquhar et al.](#_bookmark3), [1980](#_bookmark3); [von Caemmerer](#_bookmark9), [2000](#_bookmark9)) to represent how solar radiation, atmospheric CO_2_ concentration and air temperature co-limit photosynthesis. We assume that the Farquhar model parameters are universal. This assumption is not valid, so this must be seen as a rough approximation of the environmen- tal controls on photosynthesis. Using the Farquhar model can increase the robustness of the model’s predictions ([Higgins et al.](#_bookmark7), [2020](#_bookmark7)), suggesting that it is better to use this uni- versal Farquhar approximation than trying to estimate the environmental dependencies of carbon assimilation from the data. The Farquhar model photosynthetic rates are rescaled to

[0,*a_max_*] to yield *a_fqr_*. The effects of soil moisture (*M* ) on photosynthesis is represented as an increasing step function S(*M, θ*_1_*, θ*_2_) = max {min ( *M −θ*1 *,* 1) *,* 0}. This allows us to redefine

*θ*_2_*−θ*_1_

728 *a* as,

*a* = *a_fqr_* S(*M, θ*_1_*, θ*_2_) (S15)

729 The processes influencing nitrogen availability are complex and global data products on

730 plant available nitrogen are uncertain. We therefore simply assume that nitrogen uptake

731 will vary with soil temperature and soil moisture. That is, the nitrogen uptake rate *b* is

732 assumed to have a maximum rate (*b_max_*) which is co-limited by soil temperature *T_soil_* and

733 soil moisture *M* ,

*b* = *b_max_* S(*T_soil_, θ*_3_*, θ*_4_) Z(*M, θ*_5_*, θ*_6_*, θ*_7_*, θ*_8_)*.* (S16)

734 In the above equation we have assumed that the nitrogen uptake rate is a simple increas-

735 ing and saturating function of temperature. And we have assumed that the nitrogen uptake

736 rate is a trapezoidal function of soil moisture with low uptake rates in dry soils, higher up-

737 take rates at intermediate moisture levels, and lower rates once soils are so moist as to be wa-

738 terlogged. The trapezoidal function is Z(*M, θ*_5_*, θ*_6_*, θ*_7_*, θ*_8_) = max {min ( *M −θ*5 *,* 1*, ^θ^*8*−Tmax* ) *,* 0}.

*θ*_6_*−θ*_5_

*θ*_8_*−θ*_7_

739 The previous sections describe how the assimilation of carbon and nitrogen by a plant

740 are influenced by environmental factors. The TTR model describes how these assimilate

741 concentrations influence growth (equations [S3](#_bookmark4) and [S4](#_bookmark5)). In our implementation we addi-

742 tionally allow the growth rate to be influenced by temperature and moisture as it is well

743 established that cell elongation and division are influenced by temperature and moisture.

744 To mimic these effects on growth we assume that growth rate is co-limited by soil moisture

745 *M* and soil temperature *T_soil_*,

*g* = *g_max_* Z(*T_soil_, θ*_9_*, θ*_10_*, θ*_11_*, θ*_12_) S(*M, θ*_13_*, θ*_14_)*.* (S17)

746 We use *T_soil_* since we assume that growth is more closely linked to soil temperature which

747 varies slower than air temperature. Note that for simplicity, we assume that the root growth

748 and shoot growth rates are the same and that they are both influenced by temperature and

749 moisture in the same way; this assumption although not true ([Koerner](#_bookmark0), [2003](#_bookmark0); [Larcher](#_bookmark1), [2003](#_bookmark1))

750 is a pragmatic simplification.

751 The biomass loss rate (*d*, equations [S5](#_bookmark6) and [S6](#_bookmark8)) decreases as a function of soil moisture

752 (*M* ),

*d* = *d_lo_* (1 + *θ*_17_(1 *−* S(*M, θ*_15_*, θ*_16_))) *.* (S18)

753 The parameter *d* is best interpreted as a biomass loss rate that is potentially higher with soil

754 moisture levels are low, thereby allowing the model to mimic a drought deciduous strategy.

755 The parameter *θ*_17_ can be interpreted as describing the level of deciduousness.

756 **S2.1 Environmental forcing data**

757 For environmental forcing we use the ERA5-Land data ([Hersbach et al.](#_bookmark0), [2020](#_bookmark0); [Muñoz-Sabater](#_bookmark1)

758 [et al.](#_bookmark1), [2021](#_bookmark1)). The ERA5 products are global reanalysis products based on hourly estimates

759 of atmospheric variables and extend from present back to 1950. The data products are

760 supplied at a variety of spatial and temporal resolutions, we used the monthly averages

761 from 1950 to 2022 at a 0.1 x 0.1 degree spatial resolution (ca. 11 x 11 km). The ERA5-

762 Land data provide air temperature (2 m surface air temperature), soil temperature (0-7

763 cm soil depth), surface solar radiation and volumetric soil water (0-7 cm soil depth). An-

764 nual historical atmospheric CO_2_ concentrations were taken from ISIMIP (https://esg.pik-

765 potsdam.de/projects/isimip/). Each of the three study sites were in separate ERA5-Land

766 grid cells, that is the analysis of each sites phenology time series uses a different ERA5-Land

767 environmental forcing dataset.

# 768 S3 Parameter estimates

769 S3 lists the posterior estimates of the plant growth model parameters for each of the 18

770 species. The parameter names are the greek letters used in equations 1-7 and equations

771 S1-S17.

Table S3.1. Listing of the parameters in the state space model and the parameter estimates for each of the 18 study species. The main text provides details.

|  | acaexu | acager | acagra | acanig | acanil | acator | balmau | comapi | comher |
| --- | --- | --- | --- | --- | --- | --- | --- | --- | --- |
| *θ*_1_ | 7.18 | 1.45 | 0.75 | 8.69 | 5.38 | 0.82 | 1.51 | 3.16 | 0.10 |
| *θ*_2_ | 6.36 | 39.51 | 44.03 | 4.48 | 5.15 | 44.42 | 31.27 | 11.06 | 49.05 |
| *θ*_3_ | 2.86 | 18.86 | 20.09 | 8.34 | 17.80 | 13.92 | 13.04 | 14.44 | 16.54 |
| *θ*_4_ | 48.31 | 1.21 | 0.67 | 44.71 | 5.15 | 9.35 | 25.23 | 45.15 | 2.46 |
| *θ*_5_ | 3.94 | 2.48 | 5.92 | 0.89 | 5.37 | 1.21 | 3.67 | 3.47 | 1.40 |
| *θ*_6_ | 3.83 | 44.85 | 20.67 | 24.27 | 30.67 | 24.83 | 13.54 | 7.01 | 27.63 |
| *θ*_7_ | 17.20 | 21.28 | 2.45 | 0.56 | 23.86 | 3.61 | 26.26 | 36.13 | 1.67 |
| *θ*_8_ | 0.20 | 23.01 | 2.20 | 0.29 | 30.93 | 0.77 | 25.81 | 27.94 | 2.87 |
| *θ*_9_ | 17.67 | 7.94 | 19.13 | 22.69 | 19.71 | 19.02 | 19.55 | 23.54 | 23.77 |
| *θ*10 | 44.25 | 11.78 | 8.84 | 0.05 | 14.16 | 11.25 | 24.27 | 2.01 | 0.03 |
| *θ*11 | 28.18 | 27.67 | 25.22 | 23.10 | 24.17 | 28.38 | 23.41 | 29.35 | 28.23 |
| *θ*12 | 32.75 | 30.69 | 32.24 | 22.45 | 32.06 | 20.99 | 24.65 | 26.99 | 22.00 |
| *θ*13 | 0.47 | 4.95 | 10.71 | 10.48 | 9.04 | 10.97 | 11.16 | 9.44 | 9.10 |
| *θ*14 | 45.68 | 6.17 | 1.45 | 5.64 | 28.54 | 1.01 | 1.21 | 13.19 | 8.39 |
| *θ*15 | 7.71 | 5.43 | 4.74 | 10.98 | 5.10 | 4.06 | 6.33 | 11.17 | 5.41 |
| *θ*16 | 2.80 | 4.66 | 4.46 | 0.00 | 4.84 | 6.40 | 3.74 | 0.01 | 3.72 |
| *θ*17 | 42.17 | 44.87 | 48.61 | 69.92 | 45.99 | 44.45 | 47.19 | 71.71 | 44.66 |
| *x*_0_ | 111.79 | 117.43 | 92.10 | 111.69 | 108.92 | 90.56 | 112.42 | 132.06 | 92.77 |
| *λflower* | 26.73 | 26.07 | 29.98 | 20.99 | 33.60 | 34.12 | 18.19 | 20.89 | 20.08 |
| *λfruit* | 16.93 | 49.27 | 16.02 | 51.73 | 15.04 | 19.03 | 6.81 | 4.67 | 4.56 |
| *βleaf* | 2.82 | 1.52 | 2.90 | 2.99 | 3.28 | 0.70 | 0.96 | 2.87 | 2.70 |
| *δleaf,*1 | 0.28 | -2.24 | -0.73 | 0.27 | 0.19 | -3.14 | -3.42 | 0.02 | -1.69 |
| *δleaf,*2 | 1.31 | 1.96 | 0.67 | 1.21 | 0.78 | 1.10 | 1.23 | 1.26 | 1.03 |
| *δleaf,*3 | 1.00 | 0.99 | 0.92 | 0.72 | 1.10 | 1.17 | 1.39 | 1.05 | 1.09 |
| *δleaf,*4 | 1.64 | 1.84 | 1.83 | 1.55 | 2.00 | 2.04 | 2.32 | 2.07 | 1.66 |
| *βfruit* | 16.08 | 38.70 | 35.29 | 5.44 | 8.08 | 31.77 | 4.53 | 5.64 | 7.83 |
| *δfruit,*1 | 21.80 | 18.12 | 22.34 | 4.19 | 10.65 | 23.34 | 7.54 | 4.48 | 5.83 |
| *δfruit,*2 | 1.49 | 2.89 | 0.57 | 0.96 | 1.73 | 0.53 | 0.84 | 1.02 | 0.87 |
| *δfruit,*3 | 1.64 | 0.79 | 0.59 | 0.57 | 2.08 | 1.18 | 0.88 | 0.80 | 0.59 |
| *δfruit,*4 | 6.31 | 4.13 | 2.88 | 1.87 | 6.07 | 3.39 | 6.29 | 2.61 | 1.45 |
| *βflower* | 18.47 | 50.88 | 37.33 | 21.59 | 22.88 | 34.40 | 22.88 | 17.43 | 15.19 |
| *δflower,*1 | 22.62 | 22.15 | 23.43 | 11.31 | 23.53 | 24.64 | 22.28 | 12.30 | 11.52 |
| *δflower,*2 | 2.19 | 2.25 | 1.22 | 0.63 | 1.71 | 1.35 | 0.48 | 0.94 | 0.95 |
| *δflower,*3 | 2.51 | 5.47 | 2.00 | 1.87 | 1.67 | 2.43 | 1.51 | 2.88 | 1.79 |
| *δflower,*4 | 6.41 | 4.15 | 6.01 | 6.64 | 6.64 | 7.42 | 6.07 | 6.79 | 3.61 |

Parameter table continued

|  | comzey | grebic | grefla | kigafr | lansch | papcap | sclbir | terser | zizmuc |
| --- | --- | --- | --- | --- | --- | --- | --- | --- | --- |
| *θ*_1_ | 5.23 | 6.11 | 5.52 | 6.49 | 0.39 | 0.16 | 0.92 | 0.53 | 4.44 |
| *θ*_2_ | 5.39 | 8.02 | 6.49 | 3.37 | 44.05 | 49.17 | 12.79 | 16.61 | 4.79 |
| *θ*_3_ | 5.74 | 6.72 | 2.98 | 21.73 | 17.58 | 19.83 | 12.61 | 7.85 | 6.74 |
| *θ*_4_ | 24.08 | 38.44 | 46.92 | 1.46 | 2.29 | 0.25 | 48.54 | 7.44 | 48.90 |
| *θ*_5_ | 10.54 | 8.97 | 0.29 | 2.12 | 2.15 | 7.85 | 0.69 | 2.80 | 0.62 |
| *θ*_6_ | 15.92 | 11.65 | 30.74 | 48.11 | 17.15 | 18.54 | 17.14 | 49.65 | 0.87 |
| *θ*_7_ | 26.37 | 24.02 | 0.71 | 29.18 | 5.79 | 6.28 | 21.95 | 27.77 | 0.76 |
| *θ*_8_ | 27.01 | 25.42 | 0.16 | 28.67 | 9.62 | 0.05 | 21.28 | 21.50 | 47.13 |
| *θ*_9_ | 18.09 | 17.38 | 20.18 | 21.75 | 20.07 | 19.33 | 23.84 | 17.67 | 19.79 |
| *θ*10 | 22.23 | 30.11 | 10.14 | 1.53 | 14.32 | 11.18 | 0.03 | 48.61 | 11.11 |
| *θ*11 | 21.40 | 24.55 | 26.51 | 28.02 | 26.55 | 29.63 | 32.25 | 26.10 | 24.03 |
| *θ*12 | 23.73 | 22.33 | 23.90 | 17.48 | 28.17 | 18.37 | 20.12 | 28.52 | 24.24 |
| *θ*13 | 10.93 | 12.64 | 9.58 | 3.91 | 11.64 | 12.63 | 10.02 | 11.29 | 9.97 |
| *θ*14 | 11.41 | 2.75 | 20.07 | 25.33 | 0.54 | 0.02 | 8.16 | 0.07 | 14.66 |
| *θ*15 | 5.21 | 4.47 | 3.80 | 4.58 | 4.52 | 11.03 | 11.08 | 4.31 | 3.87 |
| *θ*16 | 4.81 | 5.17 | 5.10 | 3.06 | 5.34 | 0.04 | 0.02 | 4.98 | 5.47 |
| *θ*17 | 46.56 | 42.87 | 45.22 | 48.85 | 46.31 | 89.78 | 61.86 | 44.11 | 49.60 |
| *x*_0_ | 80.63 | 97.38 | 104.94 | 86.43 | 101.51 | 72.36 | 95.75 | 99.95 | 109.88 |
| *λflower* | 20.23 | 18.27 | 28.00 | 13.10 | 20.25 | 13.14 | 18.10 | 26.00 | 23.33 |
| *λfruit* | 2.10 | 9.33 | 18.54 | 2.52 | 14.01 | 7.95 | 4.10 | 15.26 | 12.89 |
| *βleaf* | 5.31 | 5.29 | 4.20 | 0.96 | 4.92 | -0.07 | 6.06 | 3.07 | 2.99 |
| *δleaf,*1 | 1.58 | 0.93 | 1.31 | -2.47 | 1.21 | -4.48 | 3.77 | 0.06 | 0.56 |
| *δleaf,*2 | 1.47 | 1.37 | 1.41 | 1.16 | 0.88 | 1.61 | 0.94 | 1.11 | 1.18 |
| *δleaf,*3 | 1.03 | 1.27 | 1.04 | 1.39 | 0.91 | 1.88 | 0.65 | 0.79 | 0.75 |
| *δleaf,*4 | 1.95 | 2.48 | 1.69 | 1.62 | 1.54 | 2.51 | 1.50 | 1.38 | 1.45 |
| *βfruit* | 7.58 | 14.73 | 9.42 | 17.07 | 16.94 | 20.38 | 11.53 | 17.74 | 9.73 |
| *δfruit,*1 | 9.81 | 13.01 | 8.44 | 9.48 | 19.49 | 16.81 | 7.99 | 15.63 | 9.27 |
| *δfruit,*2 | 1.42 | 1.03 | 0.75 | 1.85 | 0.60 | 0.79 | 0.42 | 0.74 | 0.49 |
| *δfruit,*3 | 0.76 | 1.37 | 0.76 | 0.99 | 1.07 | 0.71 | 0.77 | 0.79 | 0.99 |
| *δfruit,*4 | 5.02 | 2.43 | 2.25 | 1.92 | 1.50 | 1.28 | 2.56 | 1.93 | 2.66 |
| *βflower* | 22.39 | 17.62 | 17.03 | 38.28 | 22.62 | 22.42 | 15.64 | 21.11 | 10.30 |
| *δflower,*1 | 22.03 | 15.22 | 12.88 | 20.19 | 23.91 | 16.82 | 9.90 | 19.59 | 9.21 |
| *δflower,*2 | 1.05 | 1.52 | 1.24 | 2.05 | 1.81 | 1.03 | 1.09 | 0.58 | 0.76 |
| *δflower,*3 | 2.88 | 1.22 | 1.73 | 1.38 | 2.56 | 1.73 | 1.65 | 1.61 | 2.09 |
| *δflower,*4 | 5.52 | 6.19 | 3.66 | 5.57 | 2.01 | 7.92 | 4.05 | 6.79 | 6.81 |
